# Ten-year retrospective analysis of sudden deaths in schizophrenia

**DOI:** 10.1192/j.eurpsy.2026.10514

**Published:** 2026-07-31

**Authors:** A. V. Popa, A. Teodorescu, P. S. Petric, P. I. Ifteni

**Affiliations:** 1 Faculty of Medicine, Transilvania University of Brasov; 2 Clinical Hospital of Psychiatry and Neurology of Brasov, Brasov, Romania

## Abstract

**Introduction:**

Individuals with schizophrenia have a life expectancy reduced by 10–25 years compared with the general population [1]. While much of this excess mortality is linked to chronic somatic illness, sudden and unexpected deaths remain insufficiently studied. Sudden death is defined as an unexpected natural death occurring in previously asymptomatic individuals or within one hour of acute symptoms, excluding deaths from trauma, suicide, homicide, or overdose [2].

**Objectives:**

To describe the clinical characteristics, comorbidities, treatments, and causes of sudden death among inpatients with schizophrenia over a 10-year period.

**Methods:**

A retrospective study was conducted at the Clinical Hospital of Psychiatry and Neurology Brasov, Romania, covering January 2014–December 2024. All inpatients with schizophrenia who died suddenly and unexpectedly during admission were included. Cases were identified through hospital records, and causes of death were confirmed by the Forensic Medicine Service Brasov. Ethical approval was obtained in accordance with institutional requirements.

**Results:**

During the study period, 2,242 inpatients with schizophrenia were admitted; six cases of sudden death were identified (0.27%). Patients were aged 30–75 years. Cardiovascular comorbidities were present in three cases, metabolic disease in one, and two patient had no major somatic illness.Causes of death included mechanical asphyxia due to food bolus (n=2), pulmonary thromboembolism (n=1), bronchopneumonia (n=1), myocardiosclerosis (n=1), and acute myocardial infarction (n=1). Three patients were receiving polypharmacy with benzodiazepines and/or valproate in addition to antipsychotics. Antipsychotic treatment included risperidone, clozapine, quetiapine, amisulpride, and paliperidone.

Discussion: These cases highlight the diverse causes of sudden death in schizophrenia. Cardiovascular disease was frequent, but aspiration-related deaths also occurred, consistent with prior concerns regarding dysphagia and sedation in patients treated with antipsychotics and benzodiazepines [3]. Younger patients without significant comorbidities were also affected, suggesting multifactorial risk. Preventive strategies should address both medical comorbidities and treatment-related vulnerabilities, particularly aspiration risk and sedative polypharmacy.

**Conclusions:**

In schizophrenia, sudden deaths during hospitalisation result from multiple medical causes.Prevention requires integrated psychiatric and medical care, close monitoring of treatment, and structured aspiration precautions.

**References:**

[1] Laursen TM, Nordentoft M, Mortensen PB. Excess early mortality in schizophrenia. Annu Rev Clin Psychol 2014;10:425–48.

[2] Zipes DP, Wellens HJ. Sudden cardiac death. Circulation 1998;98:2334–51.

[3] Cicala G, Barbieri MA, Spina E, de Leon J. Swallowing difficulties and dysphagia associated with antipsychotics in adults. Expert Rev Clin Pharmacol 2019;12:47–55.

**Disclosure of Interest:**

None Declared

